# FGF2 Dual Warhead Conjugate with Monomethyl Auristatin E and α-Amanitin Displays a Cytotoxic Effect towards Cancer Cells Overproducing FGF Receptor 1

**DOI:** 10.3390/ijms19072098

**Published:** 2018-07-19

**Authors:** Karolina Weronika Świderska, Anna Szlachcic, Łukasz Opaliński, Małgorzata Zakrzewska, Jacek Otlewski

**Affiliations:** Faculty of Biotechnology, Department of Protein Engineering, University of Wroclaw, Joliot-Curie 14a, 50-383 Wroclaw, Poland; karolina.weronika.swiderska@gmail.com (K.W.Ś.); anna.szlachcic@uwr.edu.pl (A.S.); lukasz.opalinski@uwr.edu.pl (Ł.O.); malgorzata.zakrzewska@uwr.edu.pl (M.Z.)

**Keywords:** protein-drug conjugates, targeted cancer therapy, basic fibroblast growth factor, drug resistance

## Abstract

In the rapidly developing field of targeted cancer therapy there is growing interest towards therapeutics combining two or more compounds to achieve synergistic action and minimize the chance of cancer resistance to treatment. We developed a fibroblast growth factor 2 (FGF2)-conjugate bearing two cytotoxic drugs with independent mode of action: α-amanitin and monomethyl auristatin E. Drugs are covalently attached to the targeting protein in a site-specific manner via maleimide-thiol conjugation and Cu(I)-catalyzed alkyne-azide cycloaddition. The dual warhead conjugate binds to FGF receptor 1 (FGFR1) and utilizes receptor-mediated endocytosis for selective internalization into cancer cells with FGFR1. The developed conjugate displays high cytotoxicity towards all tested FGFR1-positive cell lines. Most importantly, the improved cytotoxic effect of both drugs is observed for lung cancer cell line NCI-H446. The single drug-FGF2 conjugates have no impact on the viability of NCI-H446 cells, whereas the dual warhead-FGF2 conjugate selectively and efficiently kills these FGFR1 positive cancer cells. Due to the diversified mode of action the dual warhead-FGF2 conjugate may overcome the potential acquired resistance of FGFR1-overproducing cancer cells towards single cytotoxic drugs.

## 1. Introduction

Fibroblast growth factor receptors (FGFR) comprise a group of four cell surface proteins that upon activation by fibroblast growth factors (FGFs) transmit signals from an extracellular environment, leading to cell proliferation, cell motility and the inhibition of apoptosis [1]. The malfunction in FGFR signaling (caused by mutations, gene rearrangements, gene multiplication and FGFR1 overexpression) has been observed in various malignancies such as breast, prostate and lung cancer [2,3]. Up to date, only small molecule FGFR inhibitors have been introduced to the market for the treatment of FGFR-dependent cancers [4]. However, due to their mode of action small molecule inhibitors suffer from nonspecific activity towards other tyrosine kinase receptors [5]. Thus, there is urgent need for the development of selective FGFR-targeted effective anti-cancer therapies.

Antibody Drug Conjugates (ADCs), which combine therapeutic potency of small cytotoxic molecules and targeting properties of antibodies specifically binding malignant cells, are important players in the field of targeted anti-cancer therapy [6,7]. To date, over 100 ADCs are in clinical trials and four have been approved for clinical use: Brentuximab vedotin (Adcetris^®^, Seattle Genetics), ado-trastuzumab emtansine (Kadcyla^®^, Genentech), gemtuzumab ozogamicin (Mylotarg^®^, Pfizer) and inotuzumab ozogamicin (Besponsa^®^, Pfizer) [8,9,10,11]. Rapid and sustained development of ADCs requires the use of bioorthogonal, site-specific conjugation methods, allowing the attainment of homogenous products that are crucial for ADC’s safety, stability and therapeutic effect [12]. We have recently demonstrated that FGF2, a natural ligand of fibroblast growth factor receptors (FGFRs), can be used as an alternative to antibodies for the selective and efficient delivery of cytotoxic drugs into cancer cells overproducing FGFRs [13]. Here, we expand the idea of FGF-based targeted anti-cancer cytotoxic drugs by combining FGF2 with two cytotoxic compounds displaying independent modes of action. Human FGF2, a small globular protein, can be efficiently expressed as a recombinant protein and exhibits relatively high stability.

The FGF2 dual warhead conjugate is designed to allow for site-specific conjugation of each cytotoxic compound via two different conjugation strategies: Thiol-maleimide reaction and Cu(I)-catalyzed alkyne-azide cycloaddition (CuAAC). Using this approach, each conjugation step can be precisely controlled to obtain stoichiometrically defined conjugate. For the development of FGF2 dual warhead conjugate, our payloads of choice were monomethyl auristatin E (MMAE) and α-amanitin, two highly potent drugs that exert their cytotoxic action via two unrelated mechanisms. MMAE is a synthetic analog of the antineoplastic natural product dolastatin 10, which inhibits cell division by blocking polymerization of tubulin. This cytotoxic agent is widely accepted as a toxic compound in many ADCs [14,15]. α-Amanitin, mushroom toxin from the *Amanita* species, selectively binds to RNA-polymerase II of eukaryotic cells and inhibits DNA transcription. α-Amanitin was tested in preclinical studies on pancreatic carcinomas and epithelial cell adhesion molecule (EpCAM)-expressing malignancies mouse models. Its conjugates showed high antitumoral activity, and it is described as highly active in drug-resistant cells, since due to hydrophilic structure, it is not effectively removed by multi-drug resistant transporters [16]. However, as its use thus far has been very limited, there is a risk of its immunogenicity, which has not been yet tested.

Here, we describe the development of a site-specific FGF2 dual warhead conjugate combining α-amanitin and MMAE by using thiol-maleimide and Cu(I)-catalyzed alkyne-azide cycloaddition, respectively. Our results on FGFR1-positive cancer cell lines show that the conjugate is efficiently targeting cells expressing FGFR1, leading to excellent and selective toxicity due to the combined cytotoxic effect of MMAE and α-amanitin. FGF2-based dual warhead conjugate not only kills cancer cells more efficiently than single drug conjugates, but also has the potential to limit the ability of cancer cells to develop resistance to cytotoxic drugs, which is a well-known feature of various cancers [17,18].

## 2. Results

### 2.1. Dual Conjugation of α-Amanitin and Monomethyl Auristatin E to Fibroblast Growth Factor 2 (FGF2)

**Figure 1:**
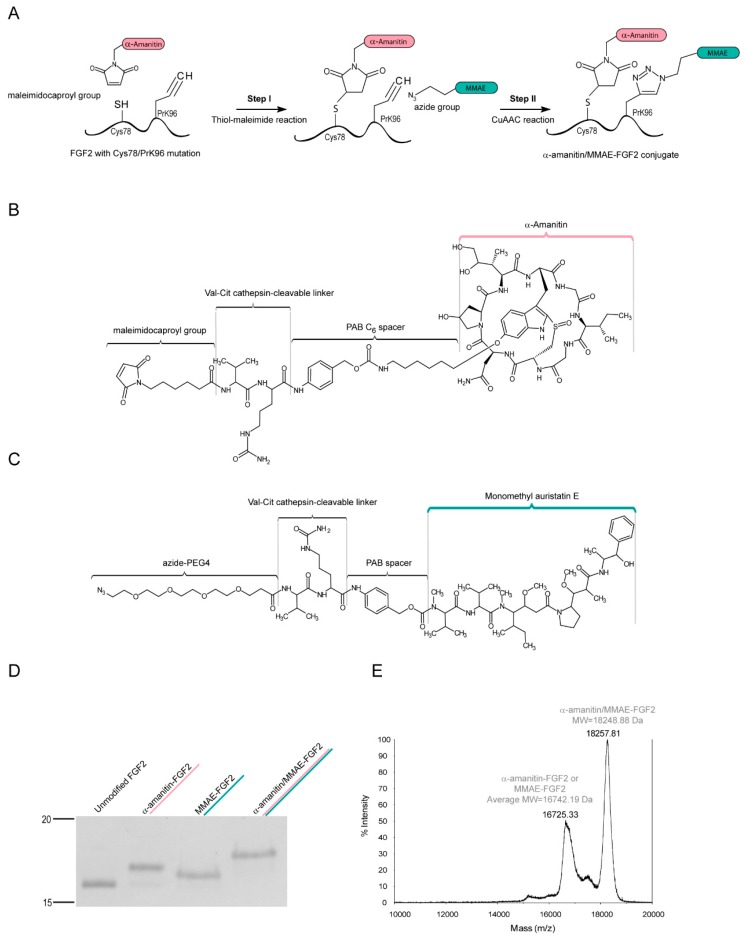
Site-specific conjugation of fibroblast growth factor 2 (FGF2) to α-amanitin and monomethyl auristatin E (MMAE). (**A**) Schematic representation of a site-specific dual conjugation by thiol-maleimide and Cu(I)-catalyzed alkyne-azide cycloaddition (CuAAC) reactions; (**B**) Chemical structure of maleimidocaproyl-Val-Cit-PAB-α-amanitin and (**C**) azide-PEG4-Val-Cit-PAB-monomethyl auristatin E; (**D**) SDS-PAGE analysis confirmed the purity of obtained conjugates; (**E**) Mass spectrometry (MS) analysis of doubly conjugated FGF2 shows attachment of one α-amanitin and one MMAE compound per one protein molecule.

The first aim of this work was the efficient production of homogenous dual warhead FGF2 conjugate (Figure 1A), with defined stoichiometry of attached maleimide-valine-citrulline-p-aminobenzyl alcohol-α-amanitin (maleimide-Val-Cit-PAB-α-amanitin) (Figure 1B) and azide-PEG4-Val-Cit-PAB-MMAE (Figure 1C) agents. In our previous studies we have optimized production of CuAAC and thiol-maleimide-based conjugates of FGF2 with single cytotoxic drugs [19,20]. Here, we chose these two different conjugation methods to allow us to independently attach two different drugs in a controlled and site-specific manner. FGF2 construct used for conjugation contained a single cysteine (Cys78) and unnatural amino acid propargyllysine (PrK) in the place of Cys96 residue. For double labeling the protein was first incubated with maleimide-functionalized α-amanitin (yielding α-amanitin-FGF2), and then the CuAAC reaction was conducted with azide-containing MMAE (resulting in α-amanitin/MMAE-FGF2). Single cytotoxic conjugates were also prepared for comparison of cytotoxic effects on cells. As shown in Figure 1D, the efficiency of both conjugation reactions is very high and has reached up to 95%, as determined by sodium dodecyl sulfate-polyacrylamide gel electrophoresis (SDS-PAGE)-based densitometry. Mass spectrometry analyses have confirmed that drug-to-protein ratio equals 1 for each drug attachment (Figure 1E).

### 2.2. Characterization of α-Amainitin/Monomethyl Auristatin E (MMAE)-FGF2 Conjugate

**Figure 2:**
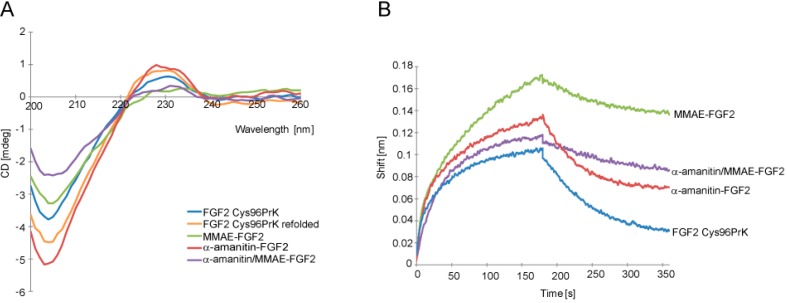
Evaluation of the secondary structure of FGF2 in conjugates and its interaction with fibroblast growth factor receptor 1 (FGFR1). (**A**) Circular dichroism (CD) spectra of native FGF2 Cys96Prk, FGF2 Cys96Prk treated with SDS as in conjugation reaction and subsequently refolded, and purified FGF2 single- and double-conjugates; (**B**) Bio-Layer Interferometry plots show binding analysis of conjugates to extracellular domain of FGFR1 (FGFR1_ECD) chemically immobilized on Amine Reactive Second-Generation biosensor (ARG2). Signal was corrected for sensor chip without immobilized FGFR1.

Next, we analyzed whether conjugation influenced structure and targeting properties of FGF2. Circular dichroism analysis revealed that protein secondary structure was preserved (Figure 2A). Since FGF2 interaction with its receptor FGFR1 is crucial for selective internalization into cells, binding of FGF2 conjugates to recombinant FGFR1 was analyzed in vitro using the bio-layer interferometry technique (BLI). All tested FGF2 conjugates retained the ability to bind to the extracellular region of FGFR1 (FGFR1_ECD) immobilized on a BLI sensor (Figure 2B) with similar value of *k*_on_ constants. For conjugates containing MMAE dissociation profiles show increased *k*_off_ constants, which may be due to the hydrophobic nature of this drug. Moreover, the conjugate cytotoxic effect is not always directly linked to its affinity to the receptor. Thus, we founded our conclusion on cytotoxic efficiency of conjugates directly on in vitro cell based assay presented below.

**Figure 3:**
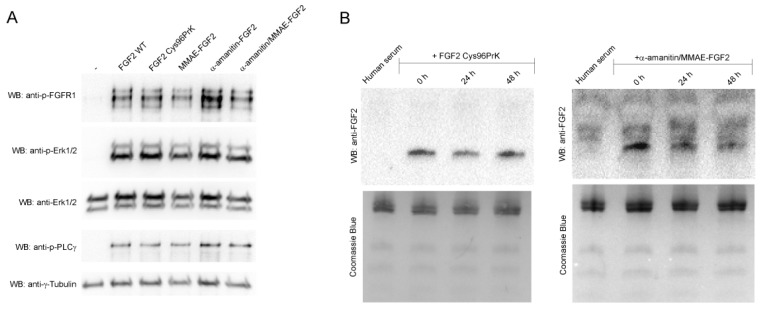
(**A**) Activation of signaling pathways in NIH 3T3 fibroblast cells. After binding of FGF3 and its conjugates to FGF receptors, FGFR1 phosphorylation and activation of downstream signaling pathways was analyzed by Western blotting; (**B**) Stability of FGF2 conjugates in the human serum. Either unmodified FGF2 or double conjugate were incubated for 24 or 48 h in full human serum at 37 °C and the amount of intact protein/conjugate was estimated by anti-FGF2 Western blotting (WB).

In order to confirm that FGF2 retained its native structure, we analyzed the ability of FGF2 conjugates to bind and activate FGFR1 present on the cell surface by studying activation of FGFR1 and downstream signaling pathways in NIH 3T3 fibroblast cells. After short-term stimulation with FGF2 or its conjugates virtually identical levels of phosphorylated FGFR1 and downstream signaling proteins: mitogen-activated protein kinases (MAP kinases) (Erk1/Erk2) and phospholipase C-γ (PLCγ) were detected (Figure 3A). For cells treated only with buffer, no downstream signaling activation was observed. 

As the overall action of conjugates relies also on the stability of used linkers, we have tested conjugates’ stability in full human serum. We did observe slightly decreased stability of double conjugate compared to unmodified FGF2 (by approximately 40% after 24 h, Figure 3B). However, as the cytotoxicity experiment setup described below includes only 2-h incubation of cells with conjugates, which are then washed away, linker stability was estimated to be sufficient.

In summary, all these data demonstrate that the addition of two various cytotoxic agents to FGF2 has not changed the FGF2 structure and its ability to interact with FGFR1 present on the cell surface.

### 2.3. FGF2 Conjugates Are Selectively Internalized into Cancer Cells in the Fibroblast Growth Factor Receptor 1 (FGFR1)-Dependent Manner

**Figure 4:**
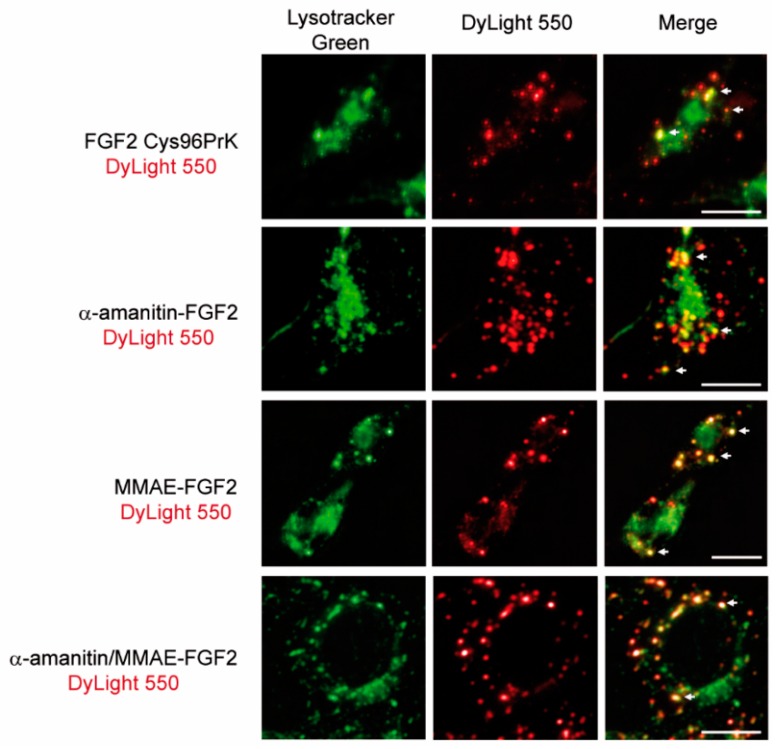
Representative images of specific colocalization of FGF2 conjugates with lysosomes. Conjugates were labeled with DyLight550 (red) and lysosomal structures were visualized with the use of Lysotracker Green (green). White arrows indicate selected regions of co-localization. Scale bars represent 20 µm.

Next, we have evaluated the targeting potential of FGF2 in the α-amanitin/MMAE conjugates. We applied fluorescence microscopy to study internalization and intracellular trafficking of conjugates into NIH 3T3 fibroblasts. FGF2 and FGF2 conjugates were labelled with amine-reactive DyLight 550 and incubated with NIH 3T3 cells. Cells were additionally stained with Lysotracker Green to visualize lysosomes, organelles where internalized conjugates should be delivered for proteolytic degradation and release of cytotoxic drugs. All tested FGF2 conjugates as well as unconjugated FGF2 were efficiently internalized into NIH3T3 cells and largely co-localized with lysosome-specific dye (Figure 4). Lysosomal delivery of protein bearing cytotoxic agents with Val-Cit linker plays a crucial role in their mechanism of action and obtained data suggest that FGF2 ensures internalization and delivery of cytotoxic drugs to lysosomes [6].

**Figure 5:**
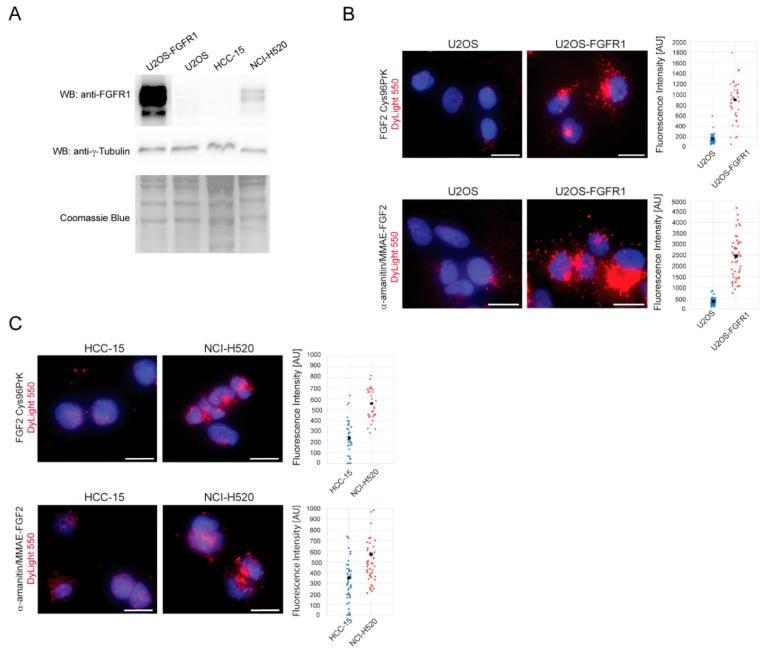
Quantitative analysis of FGF2 dual conjugate internalization into FGFR1-positive and FGFR1-negative cancer cell lines. (**A**) Western blot analysis of FGFR1 expression levels in tested cell lines. Coomassie staining was used as a loading control; (**B**) Osteosarcoma cells (U2OS) (FGFR1-negative) and U2OS-FGFR1 (FGFR1-positive) cells were treated with FGF2 dual warhead conjugate labeled with DyLight550 and intensity of fluorescence within single cells was measured and plotted. Black spots represent average fluorescence intensity (**C**) An analogous experiment was performed on squamous cell lung carcinoma HCC-15 (FGFR1-negative) and NCI H520 (FGFR1-positive). For both models, FGFR1-expressing cell lines (U2OS-FGFR1 and NCI H520) showed efficient internalization. Scale bars represent 20 µm.

Next, we assessed the FGFR1-dependence of FGF2 dual warhead conjugate internalization. We used two sets of cell lines that can be directly compared as FGFR1 positive and negative. The first set comprised of osteosarcoma cells (U2OS), characterized by a very low FGFR1 levels, together with U2OS cells stably transfected with FGFR1 (U2OS-FGFR1). For the second pair of cell lines, we chose cancer cells naturally expressing high or low FGFR levels: Squamous lung cancer cell line NCI-H520 (FGFR1 positive) compared to a non-amplified squamous lung cancer cell line HCC-15 (FGFR1 negative). Expression of FGFR1 in all tested cell lines was analyzed by Western blotting. The highest FGFR1 levels were observed in U2OS-FGFR1 cells, as these cells are stably transfected with *fgfr1* gene. NCI-H520 cells expressed moderate levels of FGFR1, whereas FGFR1 was not detected in HCC15 cells (Figure 5A).

Next, α-amanitin/MMAE-FGF2 conjugate and FGF2 control were fluorescently labeled with DyLight550, incubated with cell lines producing various amounts of FGFR1 and investigated by fluorescence microscopy. For quantitative analysis intracellular fluorescence intensity of internalized DyLight550-labelled FGF2 was measured. U2OS-FGFR1 cells, expressing high amounts of FGFR1 on their surface, internalized 5-fold higher amounts of FGF2 conjugates than control U2OS cells (Figure 5B). Squamous lung cancer cells NCI-H520 (FGFR1 positive) internalized about two times more FGF2 and FGF2 dual warhead conjugate than control, FGFR1 negative HCC-15 cells (Figure 5C). The differences in the efficiency of internalization between FGFR1 positive cell lines correlated well with the FGFR1 level on the cell surface. 

Taken together, these data demonstrate that FGF2 dual warhead conjugate is efficiently internalized into cells in FGFR1-dependent manner. Inside cells, FGF2 dual warhead conjugate is delivered into lysosomal compartments, where proteolytic degradation of Val-Cit linker may occur.

### 2.4. Cytotoxicity of α-Amainitin/MMAE-FGF2 Conjugate

**Figure 6:**
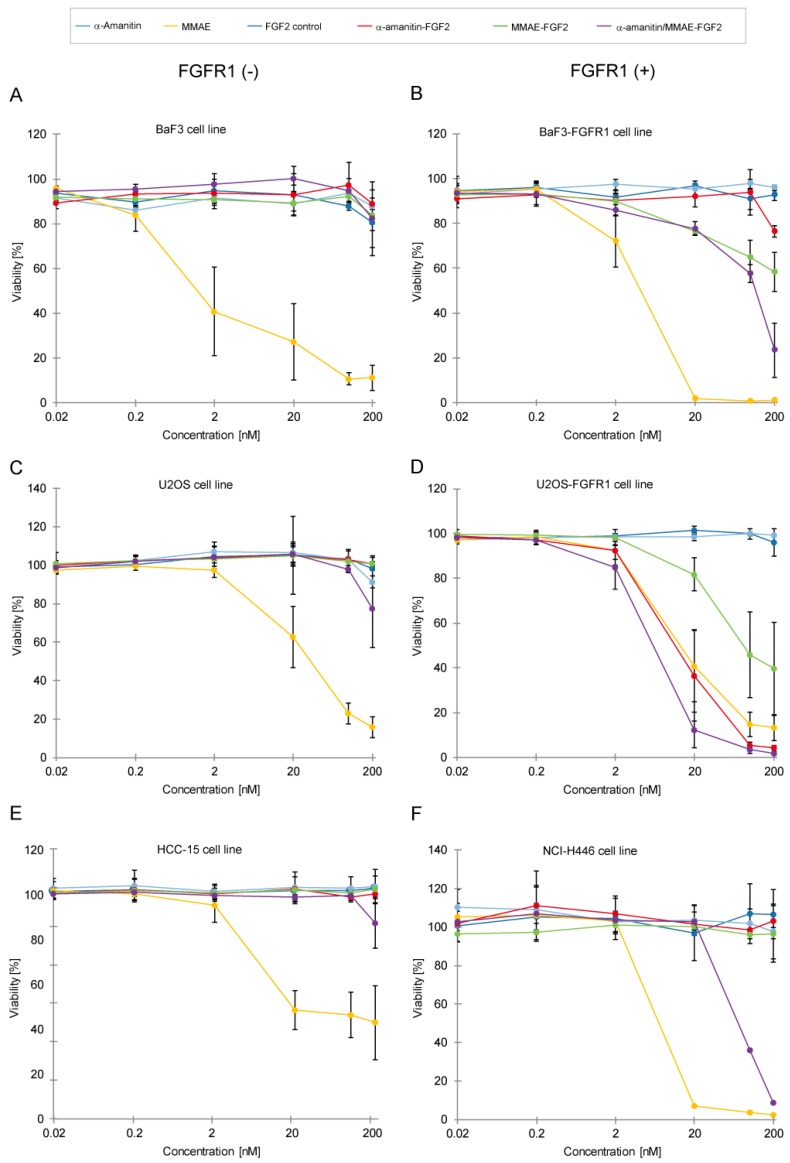
Cytotoxicity tests with FGF2 conjugates in murine pro B and cancer cell lines with different levels of FGFR1 expression. (**A**,**B**) Cytotoxic effect on BaF3 cells which represent control cell line that does not contain FGFR1 and BaF3-FGFR1 cells stably transfected *fgfr1* genes; (**C**,**D**) Conjugate incubation with osteosarcoma cell lines U2OS (FGFR1-negative) and U2OSR1 (FGFR1-positive); (**E**,**F**) Cytotoxic tests with FGF2 conjugates on lung cancer cell lines NCI-H446 expressing FGFR1 or FGFR1-negative HCC-15. Error bars represent standard deviation (SD) for *n* = 3 experiments.

The main aim of this study was to assess the cytotoxic potency of α-amanitin/MMAE-FGF2 conjugate against cancer cell lines showing overexpression of FGFR1. The selectivity and cytotoxicity of α-amanitin/MMAE-FGF2 conjugate was evaluated in in vitro studies with FGFR1-positive and FGFR1-negative cell lines (Figure 6).

First, we tested two sets of engineered model cell lines, U2OS/U2OS-FGFR1 and BaF3/BaF3-FGFR1. As described above, U2OS cells are characterized by a very low FGFR1 level in comparison with fgfr1-transfected U2OS-FGFR1 (Figure 5A). Similarly, BaF3 cells devoid of FGFR1 receptor can be directly compared to fgfr1-transfected BaF3-FGFR1. 

Each of the cell lines was treated with either unconjugated FGF2, single FGF2-drug conjugates (α-amanitin-FGF2 and MMAE-FGF2) and FGF2 dual warhead conjugate. None of tested conjugates has displayed toxicity towards receptor-negative BaF3 cells (Figure 6A). Receptor-positive BaF3-FGFR1 cells showed sensitivity to treatment with α-amanitin/MMAE-FGF2 conjugate and, to a lesser extent, to MMAE-FGF2 (Figure 6B). Similarly, U2OS cells with low FGFR1 level displayed very little response to treatment with conjugates (Figure 6C), whereas U2OS-FGFR1 cells were sensitive to each of tested FGF2 conjugates (Figure 6D). Interestingly, α-amanitin/MMAE-FGF2 had a greater cytotoxic effect than any of single-drug FGF2 conjugates, which can be explained as a result of the combined cytotoxic action of α-amanitin and MMAE. Exact IC_50_ values were not calculated due to the response of cells only to the highest concentrations of FGF2 conjugates.

We did not observe any toxicity effect of all tested conjugates for FGFR1-negative HCC-15 cell line (Figure 6E). Notably, in FGFR1-positive line NCI-H446 single FGF2 conjugates with MMAE or α-amanitin did not cause any cytotoxic effect. Only α-amanitin/MMAE-FGF2 conjugate was able to reduce cell viability by almost 95% at the highest tested concentration (Figure 6F), showing that for this cell line a concerted action of two different drugs is required to affect cancer cells viability. 

Altogether, a very low response or no response from FGFR1-deficient cell lines proves that FGF2 dual conjugate has very good selectivity towards FGFR1-positive cells. Furthermore, toxicity results correlate well with internalization experiments, confirming the FGF2-dependent specific delivery mechanism underlying toxic effects exerted by tested conjugates.

## 3. Discussion

Numerous reports show that unlike normal cells, cancer cells overproduce various isoforms of FGFRs which relate to increased cell growth, survival and angiogenesis [21,22]. FGFR-dependent cancers can be treated either with FGFRs inhibitors and/or monoclonal antibodies [23,24]. Small-molecule FGFR inhibitors, such as dovitinib (TKI258, Novartis) and nintedanib (BIBF1120, Boehringer-Ingelheim) showed promising clinical activity, but unfortunately they also exhibit nonspecific inhibitory activity towards other tyrosine kinase receptors, such as VEGFRs and PDGFRs [5,25,26]. Monoclonal antibodies raised against FGFRs are being intensively developed for treatment of FGFR-dependent cancers. For example, MGFR1877S (Genentech), a monoclonal antibody that targets FGFR3 has been approved for phase I clinical trials [27].

Highly specific antibodies can be conjugated with cytotoxic drugs in the ADC strategy for targeted cancer chemotherapy [28]. We have recently proposed FGF2, natural ligand of FGFRs as an alternative targeting factor for selective delivery of cytotoxic agents into FGFR1-overproducing cancer cells [13]. FGF2 is a molecule significantly more stable than FGF1, and binds to FGFR1, FGFR3 and, to a lesser extent, to FGFR2, allowing it to be a targeting molecule for a large group of FGFR-overexpressing cancers [29]. It is worth noting that human FGF2-based conjugates should cause no or minimal immunogenicity, as the ligand sequence is fully human. There have been previous reports presenting the use of other natural ligands of growth receptors, i.e., epidermal growth factor (EGF) conjugated with either small molecule tyrosine kinase inhibitors as genistein or large molecular fusions with cytotoxic double-stranded RNA or pancreatic ribonuclease [30,31,32]. However, in none of the tested approaches two distinct drugs have been used, which by complementary action can exert additional cytotoxic effect.

In our previous work we have shown that FGF2 with Cys96 residue substituted with unnatural amino acid, propargyllysine (PrK) residue, can be efficiently modified by CuAAC reaction, and it shows FGFR-specific cytotoxicity combined with MMAE [20]. Since FGF2 was substituted only with one MMAE per FGF2 molecule, the exhibited toxicity effects were relatively modest. To improve the FGF2-based conjugate and overcome potential heterogeneity and resistance of cancer cells to a single-drug treatment, we decided to design FGF2 dual warhead conjugate with two mechanistically different cytotoxic agents: MMAE and α-amanitin. We have obtained this site specific, homogenous conjugate using two different conjugation chemistries: Thiol-maleimide reaction (at position 78) and CuAAC protocol (at position 96). This natively folded and fully functional FGF2 dual warhead conjugate was selectively internalized into cells in FGFR1-dependent manner and co-localized with lysosomes, where the cleavage of Val-Cit linker and cytotoxic agents release occur. The FGF2 dual warhead conjugate displayed highly cytotoxic properties towards FGFR1-positive cell lines, while having no impact on the viability of FGFR1-negative control cells. 

Drug resistance of cancer cells results from a variety of factors and can lead to dramatic decrease in the efficiency of both traditional and targeted therapies [33,34]. Resistance of cancer cells can be either intrinsic or acquired over the course of treatment, and cellular mechanisms behind this phenomenon are largely elusive. Overcoming drug resistance poses a major challenge in cancer research and one of the strategies to tackle this problem is to use combinations of drugs with different modes of action. We decided to develop FGF2 dual warhead conjugate as potential therapeutic agent for treatment of drug-resistant cancers overproducing FGFRs. The greatly improved cytotoxic action of FGF2 dual warhead conjugate is perfectly illustrated by the example of small cell lung cancer line NCI-H446. NCI-H446 cells display resistance to drugs commonly used in chemotherapy: cisplatin and etoposide. The drug resistance of NCI-H446 cells is at least partially mediated by overexpression of Fidgetin-like 1 (FIGNL1) that is involved in DNA double-strand repair [35]. We didn’t observe any cytotoxic effect of single conjugates towards NCI-H446 cells, while FGF2 dual warhead conjugate killed almost 95% of cancer cells, demonstrating that dual conjugation strategy may help in combating cancer resistance. 

Site-specific dual modification remains a challenge in bioconjugation techniques. Recently, a first dually labeled Fab fragments with auristatin-based tubulin polymerization inhibitors were reported [36]. This strategy involved selective cysteine protection to achieve labeling with two drugs. Similarly, a recent report on anti-CD30 antibody labeling explored the idea of dual modification of therapeutic proteins. To obtain anti-CD30 antibody conjugate maleimide-containing multiplexing carrier with two drugs (MMAE and MMAF) was constructed that was subsequently attached to a single cysteine on the antibody [37]. Here, we developed novel and highly efficient strategy for site specific conjugation of two different cytotoxic drugs into targeting protein—FGF2. The developed FGF2 dual warhead conjugate contains two different cytotoxic agents per protein: MMAE and α-amanitin in a defined quantity and at desired location within FGF2 molecule. This is in contrast to conventional ADCs, which use only one cytotoxic compound in varying amounts per antibody molecule [12]. This is noteworthy, as the proposed dual conjugation strategy allows for attachment of any two drugs of choice, conjugates can be tailored to overcome resistance of cancer towards particular drugs. 

**Figure 7:**
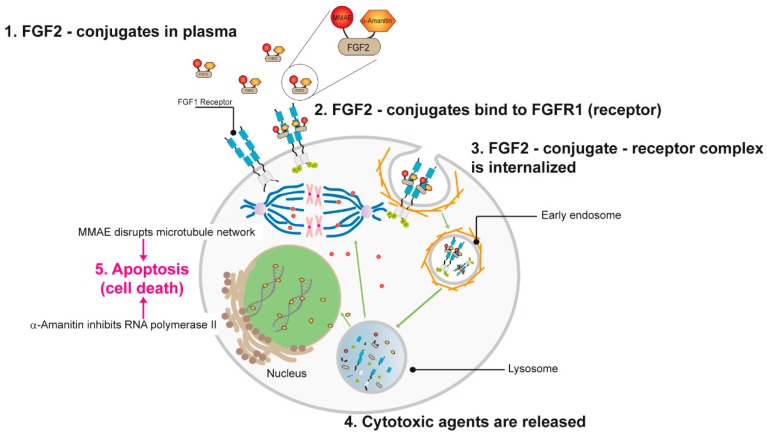
Proposed mechanism of action for α-amanitin/MMAE-FGF2 conjugate. After binding to the high affinity FGFRs on the cancer cell surface dual FGF2 conjugate is internalizing by endocytosis. Processing through the endosome–lysosome pathway leads to release of MMAE and α-amanitin inside the cell and next respectively inhibit tubulin polymerization and DNA transcription.

In summary, we propose the following mode of action for FGF2 dual warhead conjugate: The conjugate recognizes elevated levels of FGFR1 on the surface of cancer cells and utilizing receptor-mediated endocytosis it is targeted into lysosomes, degraded releasing α-amanitin and MMAE. Next, both cytotoxic agents work independently but simultaneously, inhibiting DNA transcription (α-amanitin) and preventing microtubule polymerization (MMAE) (Figure 7). Additionally, MMAE due to its ability to cross cell membrane can be exported out of the cell via multi-drug resistance transporters and extend its killing action to neighboring cancer cells, a mechanism described as the bystander effect [37]. The combination of two different cytotoxic agents together with specific targeting properties of FGF2 allowed us to establish highly potent and unique conjugate that was validated in cellular models. 

## 4. Materials and Methods 

### 4.1. FGF2 Cys96PrK Variant Expression and Purification

Human fibroblast growth factor (FGF2) Cys96PrK variant with unnatural amino acid—propargyllysine (PrK) was expressed and purified as previously described [20]. As in the previous studies, the short FGF2 form with 22 N-terminal residues truncated was used.

### 4.2. FGF2 Cys78-α-Amanitin Conjugate Preparation

Since conjugation with MMAE via CuAAC reaction involved the solubilization of resulting conjugate pellet with 0.1% SDS, and thus subsequent FGF2 refolding, to eliminate variation that could have been introduced by the additional denaturation/refolding process, all prepared FGF2 conjugates were subjected to this step. Thiol-maleimide reaction with FGF2 Cys96PrK variant (20 µM, 10 nmol) and maleimid caproyl-Val-Cit-PAB-α-amanitin (5 equiv, 100 µM, 50 nmol; Levena Biopharma, San Diego, CA, USA) was performed in 10 mM phosphate buffer pH 6.8 with 100 mM NaCl for 60 min at room temperature. After this time, l-cysteine (10 equiv, 500 nmol) was added and reaction was incubated for 30 min. Afterwards reaction mixture was centrifuged at 4 °C, 10,000× *g* for 5 min and the resulting supernatant was incubated with 0.01% SDS for 60 min at 24 °C. Reaction mixture was then centrifuged at 13,000× *g* for 5 min to remove any aggregated by-products. α-Amanitin-FGF2 conjugate was resuspended in 2.5 M guanidine hydrochloride, diluted 25-fold in 20 mM Tris-HCl, 500 mM NaCl, 1 mM ethylenediaminetetraacetic acid (EDTA) pH 7.4 and bound to Heparin Sepharose resin in suspension. After 60 min incubation at room temperature the resin was washed twice with 20 mM Tris-HCl, 500 mM NaCl, 1 mM EDTA pH 7.4 buffer and eluted with 20 mM Tris-HCl, 2 M NaCl, 1 mM EDTA pH 7.4.

### 4.3. FGF2 PrK96-MMAE Conjugate Preparation

The FGF2 Cys96PrK variant (20 µM, 10 nmol) was reacted with azide-PEG4-Val-Cit-PAB-monomethyl auristatin E (Levena Biopharma, San Diego, CA, USA) in Cu(I)-catalyzed azide-alkyne 1,3-dipolar cycloaddition. The CuAAC reaction included azide-PEG4-Val-Cit-PAB-monomethyl auristatin E (200 µM, 100 nmol), copper(II) sulfate (200 µM, 0.1 µmol), tris(3-hydroxypropyltriazolylmethyl)amine (THPTA, 1 mM, 0.5 µmol), freshly prepared sodium ascorbate (5 mM, 2.5 µmol), 5% DMSO and 0.01% SDS was performed for 60 min at 24 °C. The reaction mixture was centrifuged at 13,000× *g* for 5 min and pellet containing FGF2 conjugate was resuspended in 2.5 M guanidine hydrochloride, diluted 25-fold in 20 mM Tris-HCl, 500 mM NaCl, 1 mM EDTA pH 7.4 and loaded onto Heparin Sepharose. After a 60 min incubation at room temperature the resin was washed twice with 20 mM Tris-HCl, 500 mM NaCl, 1 mM EDTA pH 7.4 and MMAE-FGF2 was eluted with 20 mM Tris-HCl, 2 M NaCl, 1 mM EDTA pH 7.4.

### 4.4. FGF2 Cys78-α-Amanitin/PrK96-MMAE Conjugate Preparation

To prepare FGF2 Cys78-α-amanitin/PrK96-MMAE conjugate we combined described above thiol-maleimide (4.2) and CuAAC reaction (4.3) protocols. First, the FGF2 Cys96PrK variant was reacted with maleimid caproyl-Val-Cit-PAB-α-amanitin. When reaction was complete, l-cysteine was added and reaction was incubated for 30 min. Solution was centrifuged at 4 °C, 10,000× *g* for 5 min. Next, azide-PEG4-Val-Cit-PAB-monomethyl auristatin E was added and CuAAC reaction in presence of 0.1% SDS was performed. Mixture was centrifuged at 13,000× *g* for 5 min. α-Amanitin/MMAE-FGF2 pellet was resuspended in 2.5 M guanidine hydrochloride, diluted 25-fold in 20 mM Tris-HCl, 500 mM NaCl, 1 mM EDTA pH 7.4 and loaded onto Heparin Sepharose. After 60 min incubation at room temperature the resin was washed twice with 20 mM Tris-HCl, 500 mM NaCl, 1 mM EDTA pH 7.4 and α-amanitin/MMAE-FGF2 was eluted with 20 mM Tris-HCl, 2 M NaCl, 1 mM EDTA pH 7.4. Identity and purity of all conjugates were confirmed by SDS-PAGE and matrix-assisted laser desorption/ionization mass spectrometry (MALDI-TOF MS). 

### 4.5. Mass Spectrometry

MS spectra ofFGF2 Cys78-α-amanitin, FGF2 PrK96-MMAE and FGF2 Cys78-α-amanitin/PrK96-MMAE conjugates were acquired on a 4800 Plus MALDI TOF/TOF (AB SCIEX) mass spectrometer in positive-ion mode within the 2000–20,000 Da range. Samples were precipitated with 13% tri-chloroacetic acid and dissolved in 0.1% trifluoroacetic acid in 50% (*v*/*v*) acetonitrile with α-cyano-4-hydroxycinnamic acid (CHCA, Sigma-Aldrich, Darmstadt, Germany) as a matrix.

### 4.6. Circular Dichroism Spectroscopy

CD spectra were acquired for 6–7 μM protein and conjugates solutions in 20 mM phosphate buffer pH 7.4 at 20 °C in 200–260 nm range on JASCO J-815 CD spectropolarimeter.

### 4.7. BLI Assays 

Bio-Layer Interferometry technique (BLI, Octet Red K2, ForteBio^®^, Fremont, CA, USA) was used for verification of FGF2conjugates binding to ECD_FGFR1-Fc receptor [38]. Extracellular part of FGFR1 (5 µg) was chemically immobilized on Amine Reactive Second-Generation biosensors according to the AR2G Reagent Kit protocol. Measurements were performed in a kinetics buffer (ForteBio^®^) at room temperature (25 °C). Association and dissociation of the analyte α-amanitin/MMAE-FGF2, MMAE-FGF2 or α-amanitin-FGF2 conjugates (200 nM) from the ligand was monitored for 360 s. For each subsequent run, a new biosensor was prepared. 

### 4.8. The Stability of α-Amanitin/MMAE-FGF2 Conjugate in Human Serum 

Dual FGF2 conjugate or unmodified FGF2 were added to human serum (Sigma Aldrich) to a final concentration of 10 µg/mL and incubated at 37 °C for 2 days. Aliquots (20 µL) were collected for 0, 24 and 48 h. Next, samples were separated on 16% SDS-PAGE gels and analyzed by Western blotting with mouse anti-FGF2 antibodies (Santa Cruz Biotechnology, Dallas, TX, USA). 

### 4.9. Western Blotting 

FGF2-induced activation of signaling cascades in NIH 3T3 cells and western blotting was performed as described [20]. Western blotting analysis was accomplished using the following primary antibodies against: phospho-FGF Receptor (55H2) (Cell Signaling Technology, Danvers, MA,USA), phospho-p44/42 MAPK (p-Erk1/2) (Cell Signaling Technology), p44/42 MAPK (Erk1/2) (Cell Signaling Technology), phospho-PLC γ1 (Tyr 783) (Santa Cruz Biotechnology) and γ-Tubulin (Sigma). To assess the level of FGFR1 expression in U2OS, U2OSR1, HCC-15 and NCI-H520 cell lines whole-cell lysates were used and probed with anti-FGFR1 antibody (9740, Cell Signaling Technology). Detection was performed with HRP-conjugated secondary antibodies and the ECL reagent (Thermo Fischer Scientific, Waltham, MA, USA) according to the manufacturer’s protocol. 

### 4.10. Fluorescence Microscopy

In order to confirm the specific internalization and colocalization, serum starved NIH3T3 cells were incubated for 2 h with FGF2 Cys78/PrK96 protein, α-amanitin-FGF2, MMAE-FGF2 or α-amanitin/MMAE-FGF2 conjugate, all labeled with amine-reactive DyLight550 according to manufacturer’s protocol (Thermo Fisher Scientific) and then stained for 5 min with LysoTracker Green dye (Thermo Fisher Scientific). Wide-field fluorescence microscopy was performed using a Zeiss Axio Observer Z1 fluorescence microscope (Zeiss, Oberkochen, Germany). Images were taken using a LD-Plan-Neofluar 40/0.6 objective and Axiocam 503 (Zeiss, Oberkochen, Germany). The fluorescence of DyLight550 was visualized with a 540/552-nm bandpass excitation filter and a 575/640-nm bandpass emission filter. LysoTracker signal was visualized with a 450/490-nm bandpass excitation filter, and a 500/550-nm bandpass emission filter. Images were processed with Zeiss ZEN 2.3 software (for background substraction, Zeiss, Oberkochen, Germany,), Image J 1.50e (Fiji) (NIH, Bethesda, MD, USA) and Adobe Photoshop CS6 (, Adobe , San Jose, CA, USA).

To evaluate conjugates internalization, U2OSR1, U2OS, NCI H520 and HCC-15 cell lines were incubated with α-amanitin/MMAE-FGF2-DyLight 550 conjugate (5 µg/mL) and heparin (10 U/mL) for 2 h at 37 °C. First, nuclei were stained with NucBlue reagent (Thermo Fisher Scientific), according to protocol provided by the manufacturer and cells were fixed in 2% paraformaldehyde. DyLight550 signal was visualized on above and NucBlue signal with a 335/383-nm bandpass excitation filter and a 420/470-nm emission filter. For quantitative analysis the intensity of fluorescence within single cells was measured using ZEN 2.3 software in at least 30 cells.

### 4.11. Cell Lines

U2OS cells (human osteosarcoma, ATCC #HTB-96), U2OSR1 (U2OS cells stably transfected with FGFR1 gene, a kind gift of Dr. E.M. Haugsten from the Department of Molecular Cell Biology, Institute for Cancer Research, Oslo University Hospital), were cultivated in Dulbecco’s Modified Eagle’s Medium (Biowest, Nuaille, France) supplemented with 10% fetal bovine serum (Thermo Fisher Scientific, Waltham, MA, USA) and antibiotics (100 U/mL penicillin, 100 µg/mL streptomycin). For U2OSR1 cells growth media were additionally supplemented with geneticin (1 mg/mL). BaF3 cells (murine pro B cells, DSMZ) were grown in RPMI-1640 medium (Gibco, Waltham, MA., USA) supplemented with 10% fetal bovine serum (Thermo Fisher Scientific, Waltham, MA, USA), antibiotics (100 U/mL penicillin, 100 µg/mL streptomycin) and mouse interleukin 3 (IL-3, PeproTech, London, UK). Stably transfected BaF3 cells were kind gift of Professor D. Ornitz from Washington University School of Medicine (USA) and also cultivated in RPMI-1640 medium with 10% newborn bovine calf serum, geneticin (1 mg/mL), antibiotics (100 U/mL penicillin, 100 µg/mL streptomycin), β-mercaptoethanol (50 nM) and mouse interleukin 3 (IL-3, PeproTech, UK). HCC-15 cells (squamous cell lung carcinoma) were supplied by the Leibniz Institute DSMZ—German Collection of Microorganisms and Cell Cultures, and maintained in RPMI 1640 Medium (Biowest, Nuaille, France) supplemented with 10% fetal bovine serum and antibiotics (100 U/mL penicillin, 100 µg/mL streptomycin). NCI-H446 (small cell lung cancer, ATCC #HTB-171) and NCI H520 (squamous cell lung carcinoma, ATCC #HTB-182) were respectively cultivated in ATCC-formulated RPMI-1640 medium (ATCC, Manassas, VA, USA) supplemented with 10% fetal bovine serum and antibiotics (100 U/mL penicillin, 100 µg/mL streptomycin). NIH3T3 (murine embryonic fibroblasts, ATCC #CRL-1658) were grown in Dulbecco’s Modified Eagle’s Medium (Biowest, Nuaille, France) supplemented with 10% fetal bovine serum and antibiotics (100 U/mL penicillin, 100 µg/mL streptomycin). All cells were maintained in a humidified atmosphere at 37 °C and 5% CO_2_.

### 4.12. Cytotoxicity Assay

The cytotoxicity of FGF2 Cys78-α-amanitin, FGF2 PrK96-MMAE and FGF2 Cys78-α-amanitin/PrK96-MMAE conjugates was determined using the AlamarBlue Cell Viability Reagent (Thermo Fisher Scientific). The lung cancer cells, NCI-H446, HCC-15 and osteosarcoma cells U2OS, U2OSR1 were cultured at 5000–10,000 cells per well in a 96-well plate, depending on the cell line. After 24 h, tested FGF2 conjugates were added to the cells at indicated concentrations. Cell lines were exposed to the drug for 2 h and after that time the media were removed and replaced with a fresh medium. BaF3/BaF3-FGFR1 cells were cultured at 10,000 cells per well in a 96-well plate. Tested conjugates were added immediately after seeding and incubated for 96 h. 

For all cell lines, after 96 h of incubation media was removed and replaced with a fresh medium containing 10% of AlamarBlue Cell Viability Reagent. Cells were incubated for 4 h, and then the fluorescence at 590 nm was measured using an EnVision Multimode Plate Reader (PerkinElmer, Waltham, MA, USA). 

## Abbreviations

α-Amanitin-FGF2	FGF2 single conjugate with α-amanitin incorporated at Cys78 position
α-Amanitin/MMAE-FGF2	FGF2 dual warheads conjugate with α-amanitin incorporated at Cys78 position and MMAE incorporated at PrK96 position
ADCs	Antibody drug conjugates
AR2G	Amine reactive second-generation biosensor
ATCC	American type culture collection
BLI	Bio-layer Interferometry technique
CuAAC	Cu(I)-catalyzed azide-alkyne 1,3-dipolar cycloaddition
FBS	Fetal bovine serum
FGF2	Fibroblast growth factor 2
FGFR1	Fibroblast growth factor receptor 1
MMAD	Monomethyl auristatin D
MMAE	Monomethyl auristatin E
MMAE-FGF2	FGF2 single conjugate with MMAE incorporated at PrK96 position
NBCS	Newborn bovine calf serum
PrK	*N*-ε-Propargyloxycarbonyl-l-lysine
THPTA	Tris(3-hydroxypropyltriazolylmethyl)amine
